# Ceramide-CD300f Binding Inhibits Lipopolysaccharide-induced Skin Inflammation*

**DOI:** 10.1074/jbc.M116.768366

**Published:** 2017-01-10

**Authors:** Emiko Shiba, Kumi Izawa, Ayako Kaitani, Masamichi Isobe, Akie Maehara, Koichiro Uchida, Keiko Maeda, Nobuhiro Nakano, Hideoki Ogawa, Ko Okumura, Toshio Kitamura, Toshiaki Shimizu, Jiro Kitaura

**Affiliations:** From the ‡ Atopy Research Center and; § Department of Pediatrics and Adolescent Medicine, Juntendo University Graduate School of Medicine, 2-1-1 Hongo, Bunkyo-ku, Tokyo 113-8421, and; the ¶ Division of Cellular Therapy/Division of Stem Cell Signaling, The Institute of Medical Science, The University of Tokyo, 4-6-1 Shirokanedai, Minato-ku, Tokyo 108-8639, Japan

**Keywords:** inflammation, lipopolysaccharide (LPS), mast cell, neutrophil, receptor

## Abstract

LPS triggers inflammatory responses; however, the negative regulation of LPS responses *in vivo* remains poorly understood. CD300f is an inhibitory receptor among the CD300 family of paired activating and inhibitory receptors. We have previously identified ceramide as a ligand for CD300f and shown that the binding of ceramide to CD300f inhibits IgE-mediated mast cell activation and allergic responses in mouse models. Here we identify the critical role of CD300f in inhibiting LPS-induced skin inflammation. CD300f deficiency remarkably enhanced LPS-induced skin edema and neutrophil recruitment in mice. Higher levels of factors that increase vascular permeability and of factors that induce neutrophil recruitment were detected in LPS-injected skin pouch exudates of *CD300f*^−/−^ mice as compared with wild-type mice. CD300f was highly expressed in mast cells and recruited neutrophils, but not in macrophages, among skin myeloid cells. CD300f deficiency failed to influence the intrinsic migratory ability of neutrophils. Ceramide-CD300f binding suppressed the release of chemical mediators from mast cells and from neutrophils in response to LPS. Adoptive transfer experiments indicated that mast cells mediated enhanced edema in LPS-stimulated skin of *CD300f*^−/−^ mice, whereas mast cells together with recruited neutrophils mediated robust neutrophil accumulation. Importantly, administering a ceramide antibody or ceramide-containing vesicles enhanced or suppressed LPS-induced skin inflammation of wild-type mice, respectively. Thus, ceramide-CD300f binding inhibits LPS-induced skin inflammation, implicating CD300f as a negative regulator of Toll-like receptor 4 (TLR4) signaling *in vivo*.

## Introduction

The CD300, also known as leukocyte mono-immunoglobulin-like receptor (LMIR), CMRF35-like molecule (CLM), or myeloid-associated immunoglobulin-like receptor (MAIR), members modulate immune cell responses via their paired activating and inhibitory receptor functions (1–7). CD300f, also known as LMIR3 or CLM-1, is an inhibitory receptor that contains two immunoreceptor tyrosine-based inhibitory motifs (ITIMs)^3^ and a single immunoreceptor tyrosine-based switch motif (ITSM) in its cytoplasmic region (4, 7–9). CD300f is expressed in myeloid cells, including mast cells and neutrophils. We recently identified ceramide as a ligand for CD300f and demonstrated that the binding of extracellular ceramide to CD300f inhibits IgE- or ATP-mediated mast cell activation via its ITIMs and ITSM, in allergic responses or colitis, respectively, in mouse models (9, 10). Tian *et al.* (11) demonstrated that CD300f regulates the clearance of apoptotic cells by binding to surface-exposed phosphatidylserine. Engagement of CD300f with its specific antibody inhibits both myeloid differentiation factor 88 (MyD88) and Toll-interleukin 1 receptor-domain-containing adaptor-inducing interferon-β (TRIF)-mediated Toll-like receptor (TLR) signaling in human monocyte/macrophage cell lines (12–14), whereas it augments TLR4 signaling in mouse bone marrow-derived mast cell (BMMC) (8). However, the *in vivo* role of CD300f in innate immune responses remains poorly understood. Therefore, we examined whether CD300f regulated *in vivo* responses to LPS, a cell wall component of Gram-negative bacteria, which activates myeloid cells through TLR4 (15). Accumulated studies show that TLR4 plays an important role not only in infectious inflammation characterized by Gram-negative bacterial infection and sepsis, but also in non-infectious inflammation such as ischemia/reperfusion injury and neurodegenerative/neurological diseases (16, 17).

In the present study, we use LPS-induced skin inflammation models in WT and *CD300f*^−/−^ mice, demonstrating that CD300f deficiency remarkably enhances edema and neutrophil accumulation in LPS-stimulated skin. In general, tissue-resident mast cells and macrophages initiate neutrophil recruitment by releasing factors that induce neutrophil recruitment (*e.g.* macrophage inflammatory protein 2 (MIP2), keratinocyte-derived chemokine (KC), leukotriene B4 (LTB4), and mast cell proteases) in response to specific stimuli. Moreover, neutrophils recruit further neutrophils to the tissue by producing LTB4 and chemokines MIP2 and KC. On the other hand, mast cells play an important role in edema formation by releasing factors that increase vascular permeability (*e.g.* histamine and LTC4) (18–21). Here we describe the molecular mechanisms by which CD300f suppresses LPS-induced skin inflammation.

## Results

### 

#### 

##### LPS-induced Skin Inflammation Was Profoundly Enhanced in CD300f^−/−^ Mice as Compared with WT Mice

**FIGURE 1.**
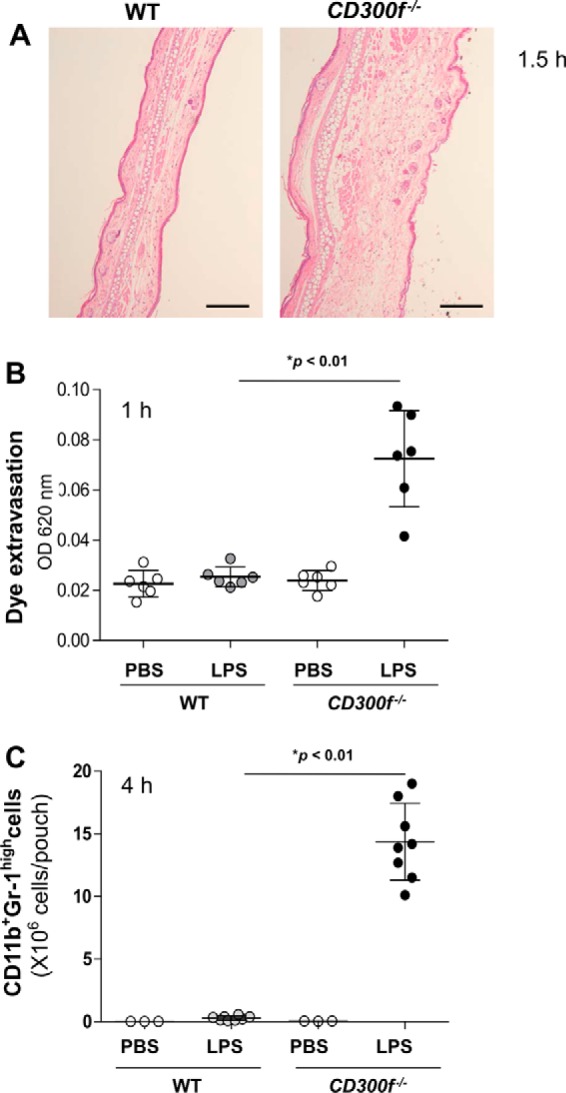
**LPS-induced skin inflammation was profoundly enhanced in *CD300f*^−/−^ mice as compared with WT mice.** *A*, hematoxylin/eosin staining of ear sections of WT and *CD300f*^−/−^ mice 1.5 h after intradermal injection with LPS (*scale bars*, 100 μm). *B*, dye extravasation in ears of WT and *CD300f*^−/−^ mice before or 1 h after intradermal injection with LPS or PBS as a control. *OD*, optical density. *C*, the number of CD11b^+^Gr-1^high^ neutrophils recruited into skin pouches of WT and *CD300f*^−/−^ mice before or 4 h after LPS or PBS injection. Data are representative of two independent experiments. Means ± S.D. are plotted. *, *p* < 0.01 (Student's *t* test).

LPS was intradermally injected into the ears of WT or *CD300f*^−/−^ mice. In histological examinations of ear sections, severe skin edema was evident 1.5 h after LPS injection in *CD300f*^−/−^ mice, but not WT mice (Fig. 1*A*) (9). Consistently, intravenous injection of Evans blue dye resulted in a massive extravasation of dye in LPS-injected ears of *CD300f*^−/−^ mice, but not of WT mice, 1 h after LPS injection (Fig. 1*B*). We next tested dorsal air pouch models of LPS-induced inflammation in WT or *CD300f*^−/−^ mice (22). We found a remarkable increase in the number of neutrophils in skin pouch exudates of *CD300f*^−/−^ mice, but not of WT mice, 4 h after LPS injection (Fig. 1*C*). Thus, CD300f deficiency enhanced edema and neutrophil recruitment in LPS-stimulated skin in mice.

##### Higher Levels of Chemical Mediators Were Detected in LPS-stimulated Skin Pouch Exudates of CD300f^−/−^ Mice as Compared with WT Mice

**FIGURE 2.**
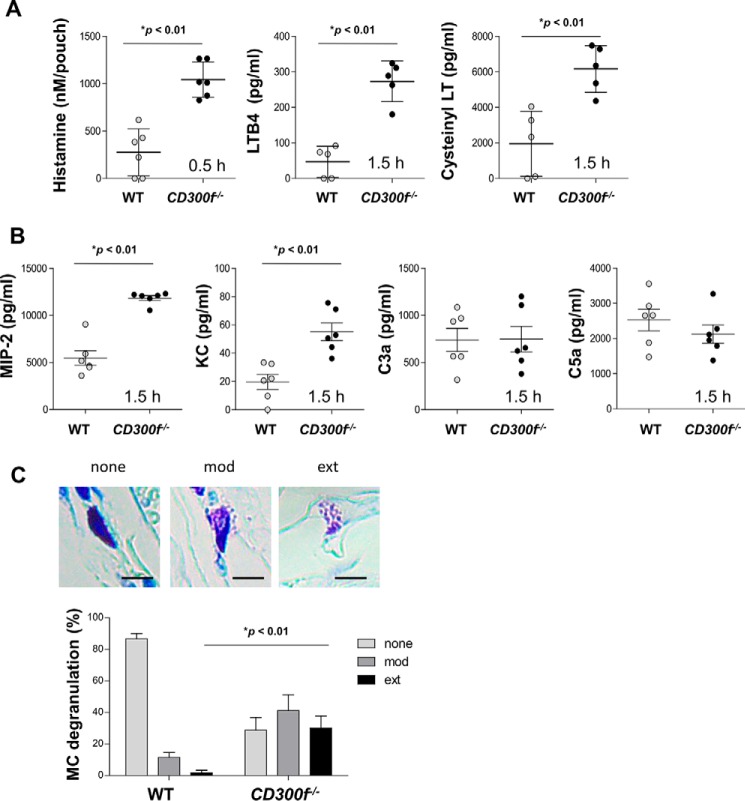
**Higher levels of chemical mediators were detected in LPS-stimulated skin pouch exudates of *CD300f*^−/−^ mice as compared with WT mice.** *A* and *B*, levels of histamine, LTB4, or cysteinyl LTs (*A*) or MIP2, KC, C3a, or C4a (*B*) in skin pouch exudates of WT and *CD300f*^−/−^ mice 0.5 h (for histamine) or 1 h (for LTB4, cysteinyl LTs, MIP2, KC, C3a, or C4a) after LPS injection. *C*, quantification of mast cell (*MC*) degranulation in LPS-stimulated dorsal skin sections of WT and *CD300f*^−/−^ mice 4 h after LPS injection was determined by classification in three categories: not degranulated (*none*: *first column*), moderately degranulated (*mod*: *second column*), and extensively degranulated (*ext*: *third column*). Percentages of mast cells belonging to each category are shown. *Insets* show degranulated (not, moderately, or extensively) mast cells in toluidine blue-stained sections (*scale bars*, 10 μm). Data are representative of two independent experiments. Means ± S.D. are plotted. *, *p* < 0.01 (Student's *t* test).

We then measured levels of factors that increase vascular permeability (*e.g.* histamine and cysteinyl leukotrienes (LTs)) and neutrophil chemoattractants (*e.g.* MIP2, KC, and LTB4) in LPS-injected skin pouch exudates of WT or *CD300f*^−/−^ mice (18–21). Levels of histamine, cysteinyl LTs, LTB4, MIP2, or KC were higher in *CD300f*^−/−^ mice than in WT mice, whereas those of complements C3a and C5a were not different (Fig. 2, *A* and *B*). Histological analysis showed that mast cells were frequently degranulated in LPS-stimulated skin sections of *CD300f*^−/−^ mice, but not of WT mice (Fig. 2*C*) (18, 20, 21). CD300f deficiency enhanced edema and neutrophil accumulation in skin treated with LPS, presumably due to local increases in factors that increase vascular permeability and neutrophil chemoattractants, respectively.

##### Mast Cells and Neutrophils Contributed to Enhanced Inflammation in LPS-induced Skin of CD300f^−/−^ Mice

**FIGURE 3.**
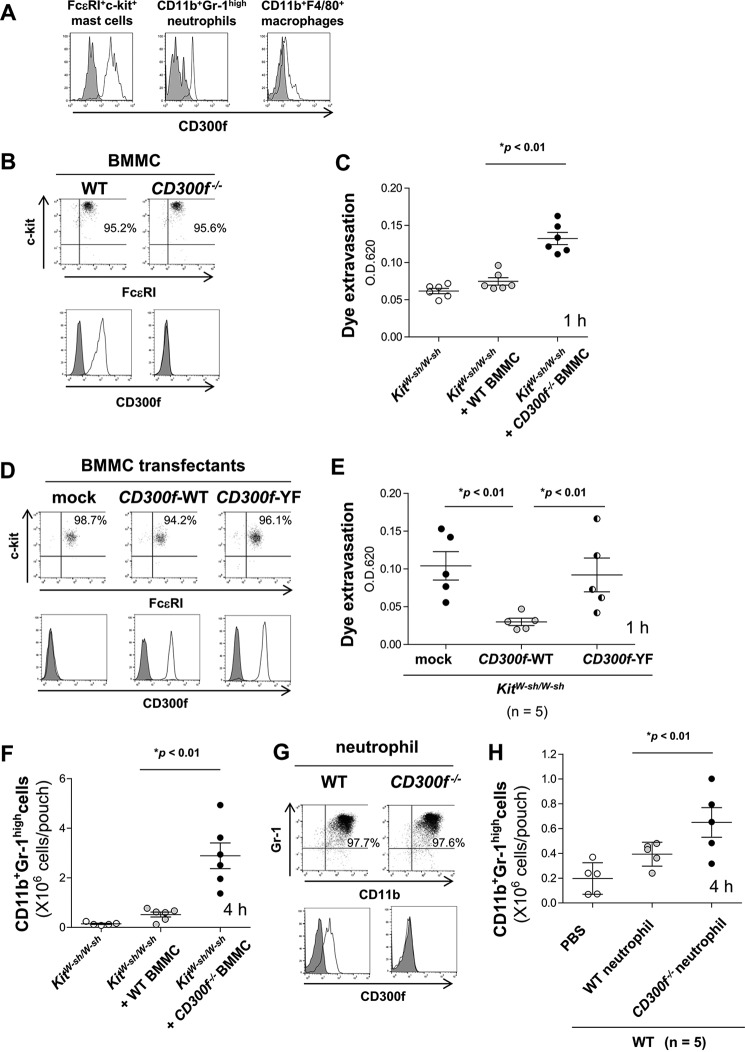
**Mast cells and neutrophils contributed to enhanced inflammation in LPS-induced skin of *CD300f*^−/−^ mice.** *A*, surface expression of CD300f in skin myeloid cells. *B*, surface expression of FcϵRI, c-Kit, or CD300f in WT or *CD300f*^−/−^ BMMC. *C*, dye extravasation in ears of *Kit^W-sh/W-sh^* mice or *Kit^W-sh/W-sh^* mice transplanted with 1 × 10^6^ of either WT or *CD300f*^−/−^ BMMC 1 h after LPS stimulation. *OD*, optical density. *D*, surface expression of FcϵRI, c-Kit, or CD300f in *CD300f*^−/−^ BMMC transduced with CD300f-WT, CD300f-Y241F/Y289F/Y325F mutant, or mock. *E*, dye extravasation in ears of *Kit^W-sh/W-sh^* mice transplanted with 1 × 10^6^ of *CD300f*^−/−^ BMMC transduced with CD300f-WT, CD300f-Y241F/Y289F/Y325F mutant, or mock 1 h after LPS stimulation. *F*, the number of neutrophils recruited to skin pouches of *Kit^W-sh/W-sh^* mice or *Kit^W-sh/W-sh^* mice transplanted with 1 × 10^6^ of either WT or *CD300f*^−/−^ BMMC 4 h after LPS stimulation. *G*, surface expression of CD11b, Gr-1, or CD300f in WT or *CD300f*^−/−^ neutrophils. *H*, the number of neutrophils (Ly5.1^+^) recruited to skin pouches of WT mice (Ly5.1^+^) transplanted with 1 × 10^6^ of either WT or *CD300f*^−/−^ neutrophils (Ly5.2^+^) 4 h after LPS stimulation. Data are representative of three (*A*, *B*, *D*, and *G*) or two (*C*, *E*, *F*, and *H*) independent experiments. Means ± S.D. are plotted. *, *p* < 0.01 (Student's *t* test).

To identify cell populations in *CD300f*^−/−^ mice that mediate the enhanced inflammatory responses in LPS-stimulated skin, we examined the surface expression of CD300f in ear skin myeloid cells. Flow cytometric analysis revealed that mast cells and neutrophils expressed high levels of CD300f, whereas other resident myeloid cells, including macrophages, expressed low or undetectable levels (Fig. 3*A*). We therefore focused on the role of CD300f in mast cells and neutrophils in LPS-induced skin inflammatory responses. To this end, we used the skin inflammation models in mast cell-deficient *Kit^W-sh/W-sh^* mice transplanted with WT or CD300f-deficient BMMC with equivalent expression levels of FcϵRI and c-Kit on the surface (Fig. 3*B*) (9, 10). Dye extravasation in LPS-stimulated ear skin of *Kit^W-sh/W-sh^* mice was enhanced by the adoptive transfer of CD300f-deficient BMMC, but not of WT BMMC (Fig. 3*C*). The following mutations in the cytoplasmic region of CD300f, Y241F, Y289F, and Y325F abolish two ITIM and a single ITSM. Vascular permeability was enhanced in *Kit^W-sh/W-sh^* mice transplanted with *CD300f*^−/−^ BMMC, transduced to express CD300f-Y241F/Y289F/Y325F, at levels comparable with those of *Kit^W-sh/W-sh^* mice transplanted with CD300f-deficient BMMC (Fig. 3, *D* and *E*) (9, 10), indicating the critical importance of the ITIM and ITSM to LPS-induced skin edema. In addition, neutrophil recruitment to LPS-stimulated skin pouches of *Kit^W-sh/W-sh^* mice was enhanced by the adoptive transfer of CD300f-deficient BMMC as compared with WT BMMC (Fig. 3*F*). On the other hand, WT or *CD300f*^−/−^ neutrophils with equivalent expression levels of CD11b and Gr-1 on the surface were injected into skin pouches of WT mice before LPS stimulation (Fig. 3*G*). Adoptive transfer of *CD300f*^−/−^ neutrophils, but not of WT neutrophils, also enhanced host-derived neutrophils recruited to LPS-stimulated skin pouches of WT mice (Fig. 3*H*). Taken together, these results indicate that CD300f-deficient mast cells play a major role in edema formation, and CD300f-deficient mast cells together with recruited neutrophils contribute to neutrophil accumulation in LPS-stimulated skin.

##### CD300f Deficiency Did Not Influence the Intrinsic Migratory Ability of Neutrophils

**FIGURE 4.**
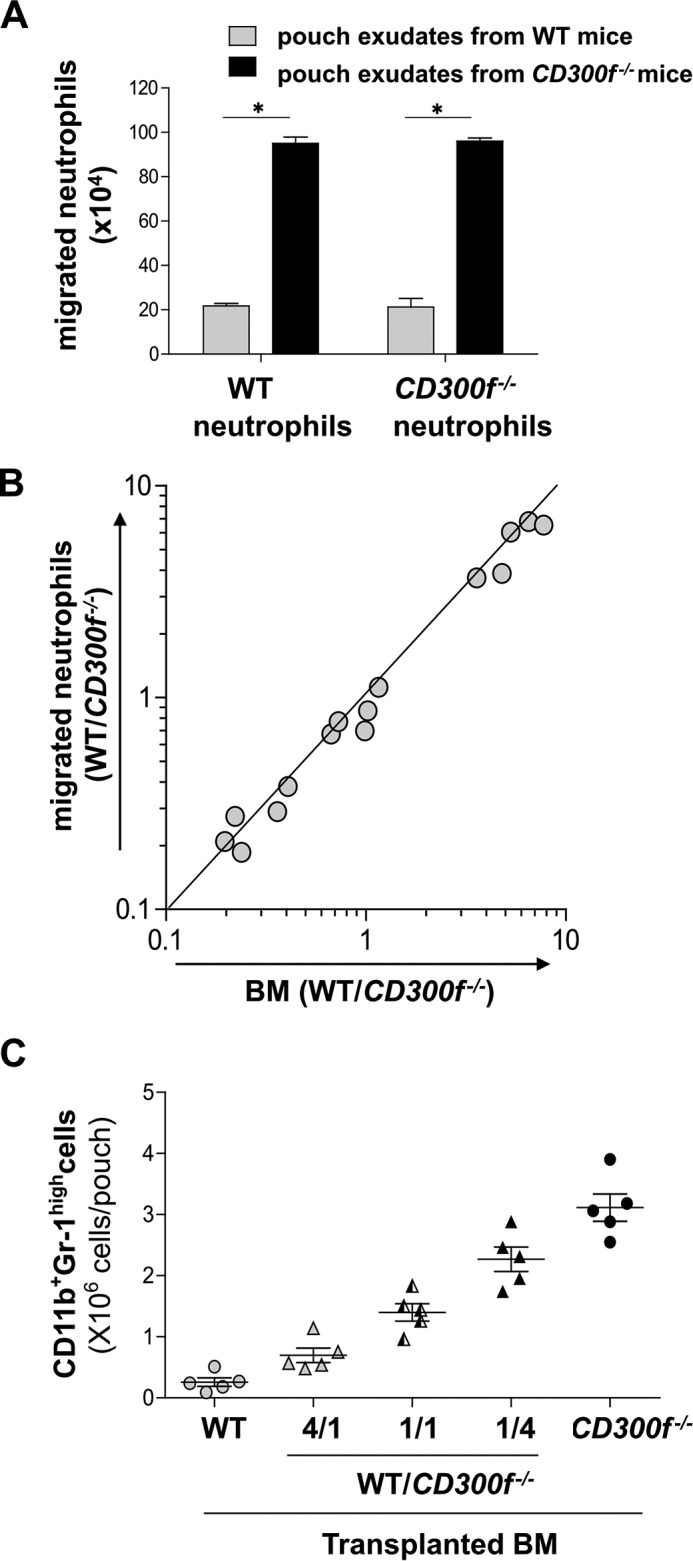
**CD300f deficiency did not influence the intrinsic migratory ability of neutrophils.** *A*, numbers of WT or *CD300f*^−/−^ neutrophils that migrated into the lower wells containing dorsal pouch exudates derived from either WT or *CD300f*^−/−^ mice 4 h after an intradermal injection of LPS. Data are representative of two independent experiments. *B*, the ratio of *CD300f*^−/−^ neutrophils in total neutrophils included in BM or dorsal pouch exudates from the mixed chimera mice (WT: *CD300f*^−/−^ = 4:1, 1:1, or 1:4) (*n* = 5) 4 h after an intradermal injection of LPS. Values for the *x* and *y* axes represent the percentage in BM and dorsal pouch exudates, respectively. *C*, numbers of total neutrophils in dorsal pouch exudates from WT BM-transplanted mice (*n* = 5), the mixed chimera mice (*n* = 5), or *LMIR3*^−/−^ BM-transplanted mice (*n* = 5) 4 h after an intradermal injection of LPS. (*B* and *C*) Data are representative of two independent experiments. Means ± S.D. are plotted. *p* < 0.01 (Student's *t* test).

Transwell migration assays demonstrated that more neutrophils were attracted to LPS-stimulated skin pouch exudates of *CD300f*^−/−^ mice, and that equivalent numbers of WT or *CD300f*^−/−^ neutrophils migrated into the same exudate (Fig. 4*A*) (22). To next examine the *in vivo* migration of WT *versus CD300f*^−/−^ neutrophils to LPS-stimulated skin pouches, we used dorsal air pouch models in mixed chimera mice that received *CD300f*^−/−^ (Ly5.2^+^) bone marrow (BM) mixed in the ratio of 1:4, 1:1, or 4:1 with WT (Ly5.2^+^) BM. We then measured accumulation of these two types of neutrophils in this model. The proportions of *CD300f*^−/−^ cells among the recruited neutrophils were similar to their proportions among BM neutrophils in all the mice 4 h after LPS injection (Fig. 4*B*). The total numbers of migrating neutrophils in the chimeric mice (*CD300f*^−/−^:WT = 1:1) were lower or higher than those in the chimeric mice (*CD300f*^−/−^:WT = 4:1) or (*CD300f*^−/−^:WT = 1:4), respectively (Fig. 4*C*). Thus, we found equivalent chemotactic abilities of WT and *CD300f*^−/−^ neutrophils in the LPS-induced skin inflammation model (22, 23). Therefore, the enhancement of neutrophil accumulation in LPS-stimulated skin pouches of *CD300f*^−/−^ mice likely depends on neutrophil chemoattractants released by both *CD300f*^−/−^ mast cells and recruited neutrophils rather than on the intrinsic migratory ability of *CD300f*^−/−^ neutrophils.

##### Ceramide-CD300f Binding Inhibited the Release of Chemical Mediators from LPS-stimulated Mast Cells and Neutrophils in Vitro

**FIGURE 5.**
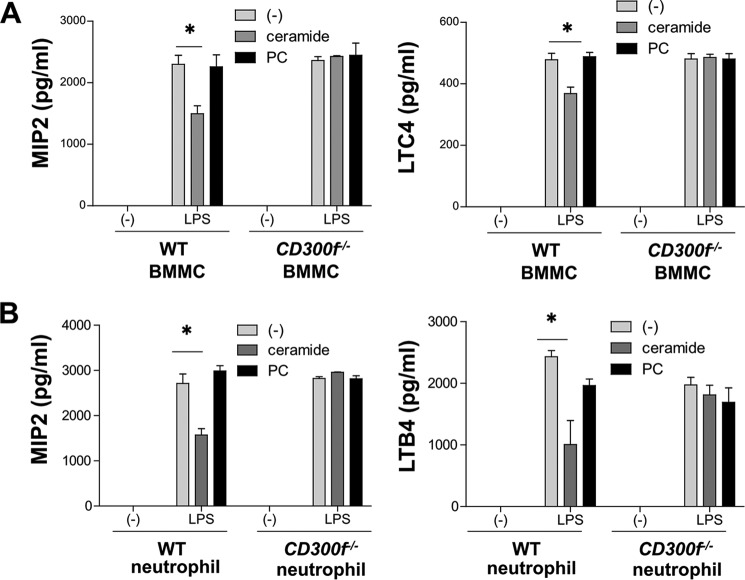
**Ceramide-CD300f binding inhibited the release of chemical mediators from LPS-stimulated mast cells and neutrophils *in vitro*.** *A* and *B*, the levels of MIP2 or LTC4 released from WT or *CD300f*^−/−^ BMMC (*A*) or neutrophils (*B*) stimulated with 100 ng/ml LPS for 6 h. Cells were preincubated on plates coated with ceramide, PC, or vehicle for 1 h before stimulation. Data are representative of three independent experiments. Means ± S.D. are plotted. *, *p* < 0.01 (Student's *t* test).

Next, we examined the effect of ceramide-CD300f binding on the release of chemical mediators from mast cells or neutrophils in response to LPS. In the absence of plate-coated ceramide, CD300f deficiency failed to influence the release of chemical mediators form BMMC or neutrophils in response to LPS. However, the binding of plate-coated ceramide to CD300f inhibited the release of MIP2 and LTC4 from LPS-stimulated BMMC (Fig. 5*A*) and of MIP2 and LTB4 from LPS-stimulated neutrophils (Fig. 5*B*) (9). Thus, ceramide-CD300f binding inhibited the release of chemical mediators from LPS-stimulated mast cells and neutrophils, implying a significant role of ceramide-CD300f interactions in innate immune responses.

##### Ceramide-CD300f Binding Inhibited LPS-induced Skin Inflammation

**FIGURE 6.**
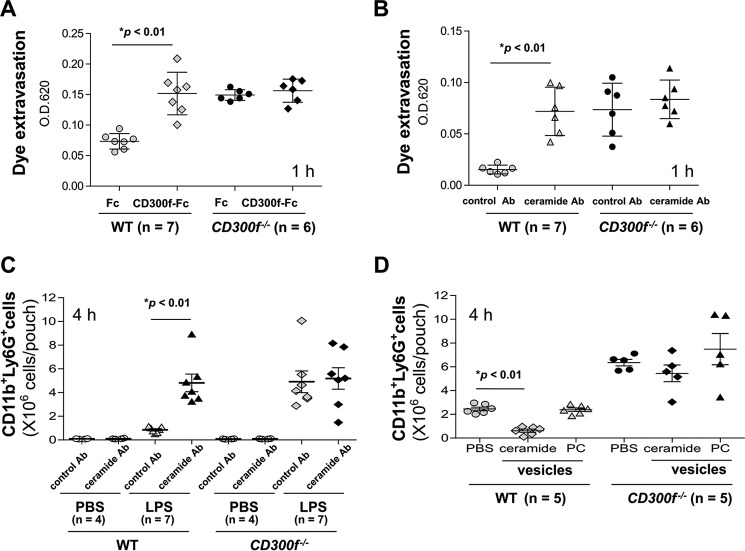
**Ceramide-CD300f binding inhibits LPS-induced skin inflammation.** *A* and *B*, dye extravasation in ears of WT and *CD300f*^−/−^ mice 1 h after intradermal injection with LPS. *OD*, optical density. *C* and *D*, the number of neutrophils recruited to skin pouches of WT and *CD300f*^−/−^ mice 4 h after intradermal injection with LPS. *A–D*, ,ice intradermally injected with CD300f-Fc or control Fc (*A*), ceramide antibody or control antibody (*B* and *C*), or vesicles containing ceramide, PC, or vehicle (*D*) 1 h before LPS injection. Data are representative of two independent experiments. Means ± S.D. are plotted. *, *p* < 0.01 (Student's *t* test).

To next address the role of ceramide-CD300f interactions in LPS-induced skin inflammation, we disrupted ceramide-CD300f binding *in vivo* with either a fusion protein, CD300f-Fc, in which the extracellular domain of CD300f was fused to the Fc domain of human IgG1, or an antibody against ceramide (9). Conversely, we increased the concentration of CD300f ligands *in vivo* by administering vesicles containing ceramide (9). Disrupting ceramide-CD300f interactions by pretreating with CD300f-Fc or ceramide antibody increased the vascular permeability of LPS-injected ear skin (Fig. 6, *A* and *B*) and the recruitment of neutrophils to LPS-stimulated skin pouches of WT mice at levels comparable with those observed in *CD300f*^−/−^ mice (Fig. 6*C*). These effects were not observed when pretreating with control Fc or antibody. In addition, pretreatment of *CD300f*^−/−^ mice with CD300f-Fc or ceramide antibody did not affect LPS-stimulated vascular permeability or neutrophil recruitment responses in skin (Fig. 6, *A–C*). Conversely, pretreatment with vesicles containing ceramide, but not with vesicles lacking ceramide, decreased neutrophil recruitment in LPS-stimulated skin of WT mice (Fig. 6*D*). Taken together, LPS-induced skin inflammation was suppressed by ceramide-CD300f binding in resident mast cells and recruited neutrophils.

## Discussion

In this study, we provide several lines of evidence that ceramide-CD300f interactions normally suppress LPS-induced skin inflammation (characterized by edema and neutrophil accumulation) by inhibiting the release of chemical mediators in LPS-stimulated skin: CD300f deficiency elevated levels of factors that increase vascular permeability and of factors that induce neutrophil recruitment in LPS-stimulated skin and remarkably enhanced skin inflammation; administering a ceramide antibody or ceramide-containing vesicles enhanced or inhibited, respectively, LPS-induced skin inflammation of wild-type mice, whereas the same treatment did not influence that of *CD300f*^−/−^ mice; and CD300f deficiency failed to influence the intrinsic migratory ability of neutrophils *in vitro* and *in vivo*.

There are several conflicting reports regarding the association between ceramide and LPS responses *in vitro*: ceramide acts as a TLR4 agonist in human epithelial cells (24), yet ceramide negatively regulates TNF-α production in mouse macrophages (25). In most cases, soluble short-chained ceramide was used as an exogenous ceramide. On the other hand, plate-coated long-chained ceramide or vesicles containing long-chained ceramide were used in our experiments. Different ceramide species might exert differing effects on LPS responses in a variety of cells.

In accordance with the finding that CD300f was highly expressed in mast cells and neutrophils, but not in macrophages among skin myeloid cells, ceramide-CD300f binding inhibited the release of chemical mediators from mast cells and from neutrophils in response to LPS *in vitro*. Given that a CD300f antibody, coated on plates, enhanced LPS-induced cytokine production in BMMC (8), the strength of CD300f aggregation induced by its ligand ceramide or by a specific antibody appears to be associated with the negative or positive regulation of LPS signaling in BMMC. In any case, adoptive transfer of *CD300f*^−/−^ mast cells, but not of WT counterparts, enhanced LPS-induced skin edema and neutrophil recruitment in mast cell-deficient mice. Because LPS administration induces mast cell degranulation *in vivo*, although LPS stimulation fails to do so *in vitro* (18, 20), it is possible that ceramide-CD300f binding suppresses LPS-induced mast cell degranulation *in vivo*. On the other hand, transfusion of *CD300f*^−/−^ neutrophils, but not of WT counterparts, enhanced LPS-induced recruitment of recipient neutrophils in WT mice. Collectively, these results indicated that mast cells play an important role in edema formation, whereas mast cells together with recruited neutrophils contribute to neutrophil accumulation in LPS-stimulated skin of *CD300f*^−/−^ mice. Moreover, it is possible to speculate that CD300f inhibits mast cell degranulation, leading to the release of histamine and mast cell proteases, as well as its production of cytokines, chemokines, and lipid mediators in LPS-stimulated skin, whereas CD300f inhibits neutrophil release of chemical mediators, including neutrophil chemoattractants. However, it should be noted that a contributory role of other *CD300f*^−/−^ myeloid cells cannot be ruled out. Thus, on the basis of previous *in vitro* studies (12–14), we clarified a novel role of ceramide-CD300f binding in LPS signaling *in vivo*.

Our *in vivo* results suggest that disrupting ceramide-CD300f interactions could promote the local recruitment of neutrophils to skin infected by Gram-negative bacteria (19). Because human CD300f binds both ceramide and sphingomyelin (26), a novel drug specifically disrupting these interactions might be a promising treatment for bacterial skin infections. Because CD300f deficiency also enhances neutrophil accumulation induced by intraperitoneal injection of LPS (data not shown), treatment with ceramide-containing vesicles might improve TLR4-dependent inflammation not only in skin but also in other tissues. However, further examination will be required to delineate the role of CD300f in human relevant diseases.

In conclusion, ceramide-CD300f interactions inhibit LPS-induced skin edema and neutrophil accumulation, implicating CD300f as a negative regulator of TLR4 signaling in myeloid cells *in vivo* that is involved in a variety of TLR4-dependent non-infectious inflammatory diseases as well as infectious diseases.

## Experimental Procedures

### 

#### 

##### Mice

All procedures were approved by the institutional review committees of The University of Tokyo (Approval Number 20-8) and Juntendo University (Approval Number 270015). C57BL/6 mice (Ly-5.1 and Ly-5.2) (Charles River Laboratories Japan), *CD300f*^−/−^ mice, and *Kit^W-sh/W-sh^* mice were used as described (8, 9, 27).

##### Cells

BMMC and transduced BMMC (more than 90% of living cells expressed both c-Kit and FcϵRI) were prepared according to the following methods, as described previously (8, 28). Neutrophils (more than 90% of living cells expressed both CD11b and Gr-1) were isolated from BM using a three-layer gradient as described previously (7, 22).

##### Antibodies and Other Reagents

The following antibodies were used: rat anti-CD300f monoclonal antibody (3-14-11; rat IgG_2a_) (ActGen); FITC-conjugated CD11b (M1/70), F4/80 (BM8), FcϵRIα (MAR-1), and Ly5.1(A20), phycoerythrin (PE)-conjugated Gr-1 (RB6-8C5), CD11b (M1/70), c-Kit (2B8), Ly5.2 (104), and rat IgG2a (eBR2a) (eBioscience); PE-conjugated Ly-6G (1A8) and streptavidin-allophycocyanin (APC) (BioLegend); anti-ceramide (MID 15B4) (Enzo Life Sciences); and anti-mouse IgM (MOPC-104E) (BioLegend). Cytokines were obtained from R&D Systems, and C-24 ceramide was obtained from Toronto Research Chemicals, Inc. 1,2-Dipalmitoyl-*sn*-glycero-3-phosphocholine (PC) was obtained from Echelon Biosciences Inc.

##### Generation of Fc Fusion Proteins

cDNA fragments corresponding to the extracellular domain of CD300f were inserted into the cloning sites of the pME18S-hIgG1 Fc vector (a kind gift from H. Arase, Osaka University) (29). Fc fusion proteins were purified as described previously (9). Endotoxin levels of Fc fusion proteins, measured using Limulus Amebocyte Lysate (Lonza), were less than 0.01 ng/μg of protein.

##### Preparation of Vesicles Containing Lipids

After 1 mg of dry lipid (C-24 ceramide or PC) was hydrated with 1 ml of PBS, vesicles were generated using an Avanti Mini-Extruder (Avanti Polar Lipids, Inc.) according to the manufacturer's instructions, as described previously (9). The extruder stand and heating block were placed on a hot plate. The plunger of the syringe containing lipid samples was pushed through the membrane with a pore size of 100 nm until the lipid solution was completely transferred to the alternate syringe. A total of at least 10 passes were performed to obtain homogeneous vesicles containing indicated lipids.

##### Cell Treatments

Lipids (C-24 ceramide or PC) were diluted to a concentration of 20 μg/ml in methanol. MaxiSorp 96-well plates (Nunc, catalogue number 430341) were coated with 50 μl of each solution, air-dried, and washed twice with medium, as described previously (9). BMMC or neutrophils were preincubated on lipid-coated plates for 1 h before stimulation with 100 ng/ml LPS for 6 h.

##### Measurement of Chemokines, Histamine, Complement Proteins, and LTs

ELISA kits for KC, MIP-2, C5a, and LTB4 (R&D Systems), histamine (MBL), C3a (BD Biosciences), and cysteinyl LTs (Cayman Chemical Company) were used (9).

##### Flow Cytometry

Flow cytometric analysis was performed with a FACSCalibur (BD Biosciences) equipped with CellQuest software and FlowJo software (TreeStar) (8).

##### DNA Constructs

The construction of pMXs-internal ribosome entry sites (IRES)-puro^r^ (pMXs-IP) and pMXs-Flag-CD300f or CD300f-Y241F/Y289F/Y325F-IP was described previously (9, 30).

##### Transfection and Infection

Retroviral transfections were performed as described previously (9, 30). Retroviruses were generated by transient transfection of PLAT-E packaging cells (31).

##### BM Transplantation

BM transplantation in mice was performed as described previously (9). Briefly, 1 day after lethal γ-irradiation and 8 weeks before experiments, recipient mice (Ly5.1^+^) were intravenously injected with a total of 3 × 10^6^ cells mixed at the indicated ratios of WT *versus CD300f*^−/−^ BM (Ly5.2^+^) cells 8 weeks before experiments. *In vivo* chemotactic ability of WT *versus CD300f*^−/−^ neutrophils was assessed as described previously (23).

##### BMMC Reconstitution and Neutrophil Transfusion

*Kit^W-sh/W-sh^* mice were injected into dorsal pouches with 1 × 10^6^ of either WT or *CD300f*^−/−^ BMMCs 6 weeks before LPS administration. WT mice were injected into dorsal pouches with 1 × 10^6^ of either WT or *CD300f*^−/−^ neutrophils 1 h before LPS administration (9). Ears of *Kit^W-sh/W-sh^* mice were intradermally injected with 1 × 10^6^ of either WT or *CD300f*^−/−^ BMMC or with 1 × 10^6^ of transduced BMMC 6 weeks before LPS administration (9).

##### LPS-induced Ear Skin Inflammation Model

Mice were intradermally injected with 10 μg of LPS or PBS to each ear 30 min before intravenous injection with 1% Evans blue dye (Sigma). The amount of extravasated dye 1 h after LPS administration was measured by absorbance at 620 nm (9). In some experiments, 10 μg of anti-ceramide (MID 15B4), isotype control, CD300f-Fc, or Fc was injected intradermally in ears 1 h before LPS administration. The doses of antibodies or Fc proteins were chosen based on previous results in mouse models of passive cutaneous anaphylaxis (9). After ears were fixed with 10% formaldehyde and embedded in paraffin, sections were stained.

##### Dorsal Skin Air Pouch Model

Air pouches were formed on the dorsal skin of mice following previously described methods (22). Briefly, 5 ml of sterile air was injected subcutaneously into the dorsal skin on days 0 and 3. On day 6, 10 μg of LPS was injected into the air pouches. At a given time after injection, the air pouches were lavaged with 1 ml of PBS. Total cells in the pouch exudates were counted 4 h after LPS administration, and the percentages of CD11b^+^Gr-1^high^ (or CD11b^+^Ly-6G^+^) neutrophils were estimated by FACS. The concentrations of chemical mediators in the pouch exudates were measured by ELISA 1 h after LPS administration. In some experiments, 10 μg of anti-ceramide (MID 15B4), isotype control, or 100 μg of vesicles containing the indicated lipids was injected into skin pouches 1 h before LPS administration. The doses of antibodies or vesicles were chosen based on previous results in mouse models of passive cutaneous anaphylaxis or colitis (9, 10).

##### Quantification of Mast Cells

Mast cells were stained with toluidine blue and quantified as described (9). Quantification of mast cell degranulation was classified as extensively degranulated (>50%), moderately degranulated (10–50%), or not degranulated (<10%), as described previously (32).

##### Transwell Migration Assays

Migration assays were performed using Transwell filters with 3-μm pores (BD Falcon), as described previously (22). Briefly, the upper wells were seeded with 1.5 × 10^6^ cells in 0.2 ml of medium, and the lower wells were filled with 0.6 ml of LPS-stimulated dorsal pouch exudates. After a 1-h incubation, the number of neutrophils that had migrated into the lower wells was counted.

##### Statistical Analyses

Results are expressed as means ± S.D. An unpaired Student's *t* test was performed to compare differences between groups.

## Author Contributions

E. S. performed all the experiments and participated in writing the manuscript. K. I., A. K., M. I., A. M., K. U., K. M., and N. N. assisted with the experiments. H. O., K. O., and T. S. analyzed the data. T. K. and J. K. conceived the project, analyzed the data, and actively participated in manuscript writing.

## Footnotes

3 The abbreviations used are:

ITIM: immunoreceptor tyrosine-based inhibitory motif
ITSM: immunoreceptor tyrosine-based switch motif
TLR: Toll-like receptor
BMMC: bone marrow-derived mast cell
KC: keratinocyte-derived chemokine
LT: leukotriene
LT: leukotriene
BM: bone marrow
PC: 1,2-dipalmitoyl-*sn*-glycero-3-phosphocholine
PE: phycoerythrin.
